# Ultrahigh Resolution fMRI at 7T Using Radial‐Cartesian TURBINE Sampling

**DOI:** 10.1002/mrm.29359

**Published:** 2022-07-04

**Authors:** Nadine N. Graedel, Karla L. Miller, Mark Chiew

**Affiliations:** ^1^ Wellcome Centre for Integrative Neuroscience, FMRIB Centre University of Oxford Oxford United Kingdom; ^2^ Wellcome Centre for Human Neuroimaging, UCL Queen Square Institute of Neurology University College London London United Kingdom

**Keywords:** 7T, fMRI, ultrahigh‐field MRI, high‐resolution fMRI, radial‐Cartesian, TURBINE

## Abstract

**Purpose:**

We investigate the use of TURBINE, a 3D radial‐Cartesian acquisition scheme in which EPI planes are rotated about the phase‐encoding axis to acquire a cylindrical k‐space for high‐fidelity ultrahigh isotropic resolution fMRI at 7 Tesla with minimal distortion and blurring.

**Methods:**

An improved, completely self‐navigated version of the TURBINE sampling scheme was designed for fMRI at 7 Telsa. To demonstrate the image quality and spatial specificity of the acquisition, thin‐slab visual and motor BOLD fMRI at 0.67 mm isotropic resolution (16 mm slab, TRvol = 2.32 s), and 0.8 × 0.8 × 2.0 mm (whole‐brain, TRvol = 2.4 s) data were acquired. To prioritize the high spatial fidelity, we employed a temporally regularized reconstruction to improve sensitivity without any spatial bias.

**Results:**

TURBINE images provide high structural fidelity with almost no distortion, dropout, or T_2_* blurring for the thin‐slab acquisitions compared to conventional 3D EPI owing to the radial sampling in‐plane and the short echo train used. This results in activation that can be localized to pre‐ and postcentral gyri in a motor task, for example, with excellent correspondence to brain structure measured by a T_1_‐MPRAGE. The benefits of TURBINE (low distortion, dropout, blurring) are reduced for the whole‐brain acquisition due to the longer EPI train. We demonstrate robust BOLD activation at 0.67 mm isotropic resolution (thin‐slab) and also anisotropic 0.8 × 0.8 × 2.0 mm (whole‐brain) acquisitions.

**Conclusion:**

TURBINE is a promising acquisition approach for high‐resolution, minimally distorted fMRI at 7 Tesla and could be particularly useful for fMRI in areas of high B_0_ inhomogeneity.

## Abstract 2

Click here for author‐reader discussions

## 1 INTRODUCTION

Methods for fMRI data acquisition are overwhelmingly based on EPI sampling strategies. The enduring popularity of multi‐slice 2D EPI readouts speaks to the robustness and efficiency of EPI and is still heavily relied upon today for acquiring T_2_*‐weighted images of the whole brain at TRs ≤2–3 s. In recent years, various extensions to multi‐slice 2D EPI have been proposed, including multi‐shot 3D EPI,^1^ echo volume imaging^2^ and simultaneous multi‐slice–EPI.^3^ These extensions all provide the opportunity for significant acceleration and reduction of volume acquisition times by extending the sampling domain across the slice (or 3D) dimensions. Of these, simultaneous multi‐slice–EPI has achieved the most significant adoption, particularly in conjunction with controlled aliasing in parallel imaging (CAIPI)‐blipping schemes to reduce noise amplification penalties,^4^ although 3D EPI has continued to be investigated for use in fMRI, particularly at 7 Tesla (7T) and above.^5^, ^6^, ^7^

Whereas acceleration can provide higher temporal and/or spatial resolution, the spatial resolution in EPI‐based methods are still limited by “echo train effects” such as distortion and T_2_* blurring (particularly in echo volume imaging) and slice excitation profiles (simultaneous multi‐slice). These effects are amplified at 7T, directly undermining the 7T potential for high‐resolution fMRI despite the SNR boost the higher field provides. However, for applications such as the examination of fine structures like cortical layers or columns, true submillimeter spatial resolution is required. To achieve this higher spatial resolution, alternative strategies need to be employed. Highly in‐plane segmented EPI can achieve this by shortening the echo train and the effective echo spacing; however, it comes with long volume acquisition times due to optimizing TEs for BOLD contrast and suffers from physiologically induced k‐space inconsistencies across shots. Keyhole‐based approaches have been combined with EPIK^8^ for high‐resolution imaging, although in this approach only the central portion of k‐space is updated, leading to blurred functional contrast as well as blurring due to the inconsistent velocity of k‐space traversal in the phase‐encoding direction.

Non‐EPI trajectories have also been used for fMRI data acquisition, most commonly using spiral readouts in 2D^9^ or 3D stacks of spirals^10^, ^11^ but also radial trajectories in 2D^12^ and 3D,^13^ 3D rosette trajectories,^14^ and 3D concentric shells.^15^ These approaches are used for their improved readout efficiency, robustness to off‐resonance effects, or ultrafast single‐shot imaging. More recently, spiral acquisitions have also been employed for high‐resolution fMRI^16^, ^17^; however, like EPI, spiral imaging can also suffer from blurring (caused by off‐resonance and T_2_* signal decay over the readout). Highly anisotropic FLASH imaging^18^ has been used to generate images with ultrahigh resolution along the readout direction (0.1 mm) but with lower resolution in the phase encode and slice directions (1.4 mm and 2.0 mm, respectively), and with limited coverage of only a single slice.

**FIGURE 1:**
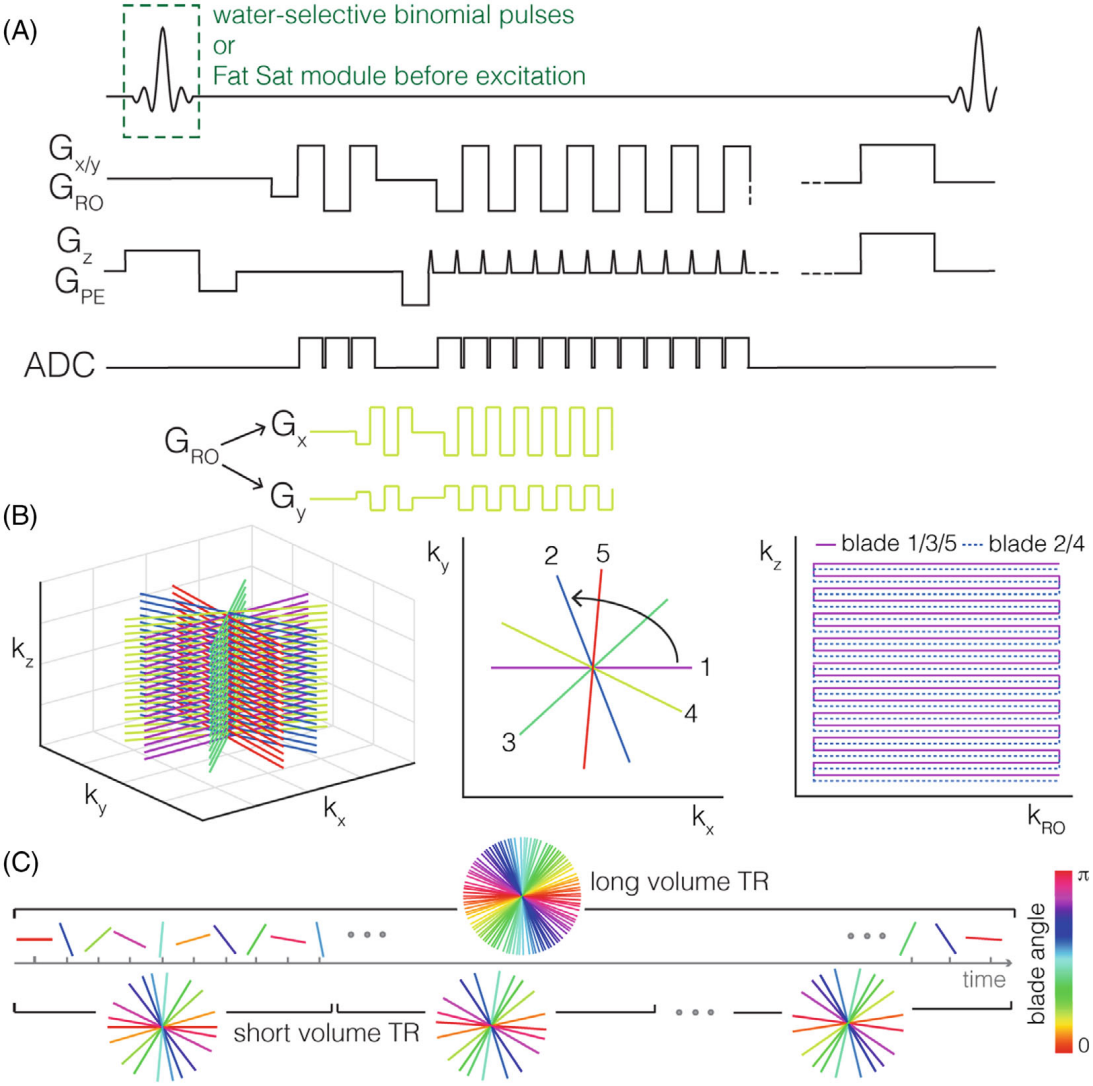
(A) TURBINE pulse sequence diagram: The gradient trajectory is the same as for standard EPI, but the angle of the readout direction changes from shot to shot, shown for 1 example (yellow) blade. Fat suppression (not shown in diagram) was achieved either via water‐selective binomial excitation or with a fat suppression module preceding excitation. (B) TURBINE trajectory: The k_xy_‐k_z_ EPI “blades” are rotated in a golden ratio angle scheme about the (phase encoding) k_z_‐axis. For acceleration within EPI blades, alternating lines are skipped for alternating blades. (C) Illustration of the flexible spatiotemporal resolution property of golden angle sampling, allowing postacquisition choice of undersampling and temporal resolution

Recently, we have investigated the use of a 3D multi‐shot radial‐Cartesian sampling strategy called *TURBINE*.^19^, ^20^, ^21^ TURBINE uses an EPI readout to sample planes of 3D k‐space, rotating successive readouts about the phase‐encoding axis to provide cylindrical k‐space coverage over multiple shots (Figure 1). Similar sampling approaches based on rotating EPI trajectories have also been published under various names.^22^, ^23^ As a 3D‐encoding method, TURBINE benefits from increased SNR efficiency, and the multi‐shot readout limits susceptibility to T_2_* blurring effects. Distortion is significantly reduced in TURBINE due to the radial encoding producing a virtually distortion‐free *x*‐*y* plane. Additionally, because the phase encoding is oriented in the typically shorter FOV superior‐inferior (*z*) direction in TURBINE, rather than the longer anterior‐posterior direction in conventional EPI encoding, distortion in the phase‐encoding direction is reduced due to the shorter echo train at the same bandwidth. The reduced echo‐train length also allows for shorter TEs, reducing dropout artifacts. Furthermore, because a central column of k‐space is traversed every shot, the sequence is self‐navigating for dynamic off‐resonance effects, ensuring more robust multi‐shot data consistency. The use of a golden angle rotation scheme^24^ provides flexibility in spatio‐temporal resolution at reconstruction time, which allows for self‐calibration of coil sensitivities^25^ or GRAPPA,^26^ in addition to providing a framework for motion robust reconstructions.^19^

In this paper, we demonstrate the application of the TURBINE acquisition scheme for ultrahigh‐resolution fMRI at 7T. We show distortion‐free 0.67 mm isotropic slab acquisitions centered on primary visual and motor cortex areas, enabling layer‐specific investigation of cortical activation, as well as a proof‐of‐principle anisotropic whole‐brain acquisition at 0.8 × 0.8 × 2.0 mm^3^ resolution.

## 2 METHODS

### 2.1 Sequence description

The TURBINE sequence (Figure 1) is a segmented 3D acquisition that uses a gradient‐echo EPI‐based readout to perform Cartesian encoding of the k_z_ axis and radial encoding of the k_x_‐k_y_ plane. This is achieved by rotating successive EPI readouts about the k_z_ phase‐encoding axis, resulting in a stack‐of‐stars sampling pattern (see Figure S3 for Point Spread Function). The rotations were governed by a golden angle scheme, incrementing by 180/phi ≈ 111.25°,^24^ where phi is the golden ratio. Undersampling factors are controlled by the number of shots per volume and the “in‐plane” reduction factor along the phase encoding direction. Total acceleration factors are stated relative to the number of data‐points needed for a fully sampled radial‐Cartesian k‐space (i.e., including the $\frac{\pi }{2}$ radial sampling overhead required to meet the Nyquist criterion at the edge of k‐space). CAIPI sampling strategies^27^ shift k‐space samples, resulting in reduced g‐factor penalty for parallel imaging. Compared to previous versions of the TURBINE sequence,^19^ the version implemented for this work uses CAIPI–style sampling in the EPI plane. In‐plane sampling patterns are shifted every shot such that, after *R* shots for an in‐plane acceleration factor of *R*, all phase‐encoding indices are visited (see right panel of Figure 1A for an example R = 2 acquisition). This is accompanied by corresponding shifts in the onset of the readout to maintain the same effective TE across all shots. The shifted lines in combination with the golden angle sampling scheme make this version of TURBINE fully self‐calibrating; that is, no dedicated reference data needs to be acquired because reference data can be formed from any subsection of the time‐series.

Prior to each EPI readout, 3 non‐phase‐encoded navigator lines were acquired for Nyquist ghost and radial trajectory correction. TURBINE is RF‐spoiled with a 117° quadratic phase increment^28^ and is gradient‐spoiled on all axes. In all cases, 100 dummy measurements preceded the beginning of data acquisition to ensure steady state conditions were achieved.

### 2.2 0.67 mm isotropic slab TURBINE

The 0.67 mm isotropic slabs were manually positioned on the calcarine fissure and hand region of the central sulcus for visual and motor cortex tasks, respectively, based on a 3‐plane localizer, and were otherwise identical. A FOV of 192 × 192 × 16 mm^3^ was prescribed with a matrix size of 288 × 288 × 24 for an isotropic resolution of 0.67 mm. Due to the short echo‐train of the slab encoding, no in‐plane acceleration was used (R = 1), resulting in a TE/TR = 23/58 ms. The slab selective RF excitation was 2.56 ms long, with a bandwidth‐time product of 16 and a flip angle of 13°, with a fat saturation module (fat‐selective excitation followed by crusher gradients) preceding it. Readout bandwidth of 964 Hz/px resulted in an echo spacing of 1.22 ms. Each time‐point consisted of 40 shots, for a nominal volume TR of 2.32 s and a total acceleration factor of $R_{0.67,\mathrm{iso}}=\frac{288}{40}\cdot \frac{\pi }{2}\approx 11.3$.

### 2.3 Anisotropic whole‐brain TURBINE

The aniostropic whole‐brain acquisitions were acquired with an in‐plane acceleration factor of R_PE_ = 2 using a 2.56 ms long slab selective, water excitation binomial RF excitation (1–2‐1 binomial pulse) with a bandwidth‐time product of 10, and a flip angle of 13°. A FOV of 192 × 192 × 132 mm^3^ was prescribed with a matrix size of 240 × 240 × 66 for an anisotropic resolution of 0.8 × 0.8 × 2.0 mm^3^. A TE/TR = 22/50 ms was achieved with a readout bandwidth of 1096 Hz/px and echo spacing of 1.02 ms. Each time‐point consisted of 48 shots, for a nominal volume TR_vol_ of 2.4 s and a total acceleration factor of $R_{0.8,\text{aniso}}=2\frac{240}{48}\cdot \frac{\pi }{2}\approx 15.7$.

### 2.4 Isotropic slab 3D EPI

Two isotropic slab conventional 3D EPI datasets were acquired to provide a comparison with the isotropic slab TURBINE data. Protocols were matched as much as possible, but parameters such as TE are necessarily mis‐matched due to fundamental differences between the readouts. Data were acquired using 2 similar protocols with slightly different TR_vol_, produced by increasing the FOV and matrix size in the *z*‐direction. The first acquisition had a TR_vol_ = 2.408 s, and 288 × 288 × 28 matrix size; whereas the second had TR_vol_ = 3.44 s, and 288 × 288 × 40 matrix size. All other parameters were fixed with readout bandwidth of 964 Hz/px, flip angle of 20°, TE/TR = 30/86 ms, R_PE_ = 4, FOV = 192 mm in‐plane, and 0.67 mm isotropic resolution.

### 2.5 Data collection

Data were acquired in 3 healthy volunteers (2 male), with informed consent in accordance with local ethics, on a whole‐body 7T Magnetom system (Siemens Healthineers, Erlangen, Germany) using a transmit/receive head coil with single channel transmit and 32 receive channels (Nova Medical, Wilmington MA, USA). Isotropic slab acquisitions were performed on all subjects, whereas whole‐brain anisotropic data were acquired in only 2 of the subjects. For structural comparison, 0.7 mm isotropic MPRAGE data were acquired in all subjects.

The functional data (isotropic slab and anisotropic whole‐brain) were acquired in 5‐min runs of a 30 s off/on task consisting of an 8 Hz modulated flashing checkerboard (visual slab), bilateral finger tapping (motor slab), or simultaneous checkerboard and fingertapping (whole brain). Head motion was mitigated using padding inside the head coil, and subjects were instructed to remain as still as possible during scanning.

### 2.6 Image reconstruction

**FIGURE 2:**
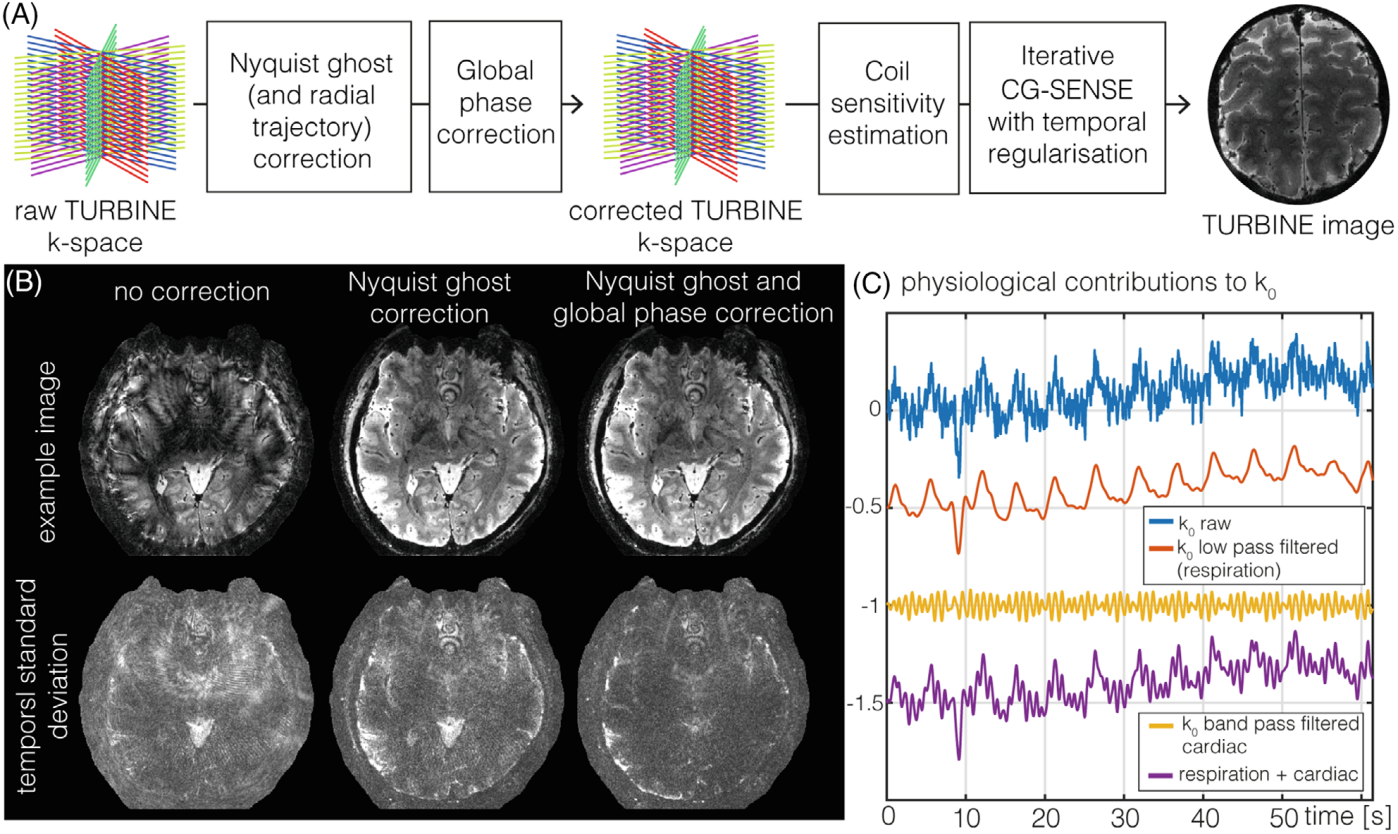
(A) TURBINE image reconstruction pipeline (B) impact of correction steps: example images and temporal SD images for no correction, Nyquist ghost correction alone, and all correction steps (C) zero order phase variation (k_0_) and filtered versions thereof to illustrate physiological contributions to k_0_

Coil sensitivities were estimated from a fully sampled temporal mean image using adaptive combine weights^29^ and then were compressed down to 12 virtual channels using singular value decomposition‐based coil compression.^30^ Several k‐space corrections were applied prior to image reconstruction (Figure 2A, left). First, a Nyquist ghost correction was applied using the 3‐line navigators to estimate the required shifts by fitting linear phase corrections to the inverse Fourier transformed navigator signals. Odd and even readout lines were each shifted by half the required offset to align all readout lines within each shot and across shots at different readout angles. This correction step simultaneously performs a radial trajectory correction because it ensures that the echoes for all blades align. Secondly, a global zero‐order phase (k_0_) correction was applied to mitigate dynamic shot‐to‐shot phase offsets due to systemic and physiological phase variations (see Figure 2C). To do this, the central column of k‐space common to every shot was extracted, and a constant phase correction term was estimated by least squares relative to a reference, in this case chosen to be the first shot. This correction was applied to all subsequent shots to align their phase to the chosen reference.^19^

The isotropic slab data is fully sampled in the EPI plane. For image reconstruction (Figure 2A, right), we therefore first performed an inverse fast fourier transform along the slab direction (*z*‐direction). Following prior work on regularized image reconstruction for fMRI time‐series data using temporal finite difference regularization,^31^ the resulting k_x_‐k_y_ slices were reconstructed using the following formulation:

$$\min_{x}{\left\|\mathrm{Ex}-k\right\|}_{2}^{2}+\lambda {\left\|\nabla_{t}x\right\|}_{2}^{2},$$

in which E is the measurement encoding operator, which incorporates non‐Cartesian k‐space sampling and coil sensitivity encoding, implemented in part using the non uniform fast fourier transform.^32^ The variable x is the estimated image time‐series; k are the noisy multi‐channel k‐space samples; λ is a regularization parameter; and $\nabla_{t}$ is the temporal finite difference operator. For the anisotropic whole‐brain data, which was acquired with undersampled EPI planes, the image reconstruction was solved as a 3D problem using the same formalism as above.

The reconstruction was implemented in Matlab 2017b (MathWorks, Natick, MA) using a conjugate gradient solver with a maximum number of iterations of 200. Regularization factors of $\lambda =10^{3},10^{4}$, and $10^{5}$ were used to evaluate reconstruction fidelity.

### 2.7 Temporal SNR

Temporal SNR (tSNR) was evaluated on the reconstructed TURBINE data with an adjusted measure that accounts for the reduced degrees of freedom (DOF) resulting from the temporal smoothing reconstruction^31^:

$$\text{tSNR}=\frac{\text{mean}(|x|)}{SD(|x|)}\sqrt{\frac{\mathrm{DOF}}{N_{t}}},$$

where the mean and SD are calculated across time, and $N_{t}$ is the number of time‐points. Because temporal smoothing introduces correlations between time points, this correction provides a more accurate estimate of the statistical power associated with the measurements, which would otherwise be inflated as smoothing decreases the temporal SD. In this case, DOF are not based on the number of reconstructed timepoints but rather are estimated using the data using Equation 9 from.^33^ In effect, the effective DOF is computed by measuring the deviation from a flat power spectrum, giving an estimate of the actual number of independent data points in the dataset after smoothing.

### 2.8 fMRI analysis

General linear model analysis was performed using FSL FEAT,^34^ including prewhitening and high‐pass filtering (filter cutoff at 60 s) but without any spatial smoothing. Minimal preprocessing was performed on the data, and no additional physiological noise correction was performed (aside from the global shot‐to‐shot phase corrections applied prior to image reconstruction). Because standard retrospective motion correction did not perform well on our very thin slab data, and to prevent interpolation‐related resolution loss, motion correction was not used (Figure S5). Gaussian‐Gamma mixture modeling^35^, ^36^ was used to ensure that the *z*‐statistic distributions are Gaussians of zero mean and unit variance and enable valid inference. The mixture modeling corrects for any loss of degrees of freedom introduced by the image reconstruction.

### 2.9 Layer‐specific analysis

To demonstrate the potential of TURBINE for laminar fMRI, we performed a simple layer‐specific analysis on 1 subject by following steps outlined by Laurentius Huber on https://layerfmri.com/2018/03/11/quick‐layering/, but using the anatomical image to determine the region of interest (ROI). The MPRAGE image was manually aligned to the subjects' functional data using the nudge tool in FSLeyes. On the MPRAGE, we identified the motor cortex in 4 slices and manually delineated an ROI corresponding to the M1‐4a subregion subregion of the hand knob, where we expect to see activation in both superficial and medial layers in a conventional finger tapping (with touch) task.^37^ We spatially upsampled the functional data by a factor 4. In the hand ROI, 20 relative cortical depths were calculated using LayNii.^38^ Activity values (*z*‐statistic scores) were pooled within each of the 20 layers.

## 3 RESULTS

The k‐space correction steps outlined in Figure 2 are essential to achieve high‐quality TURBINE images. The Nyquist ghost/trajectory correction step is especially crucial to minimize artifacts. The impact of the global phase correction on individual images is more subtle but can be seen to reduce the temporal SD. The global phase or k_0_ correction reduces fluctuations caused by physiological noise and other sources of global phase variation. Both respiratory and cardiac contributions can be clearly visible in k_0_ (especially in the low‐pass and band‐pass filtered versions) (see Figure 2C), which is sampled for each shot, that is, every 50 ms for the data shown.

**FIGURE 3:**
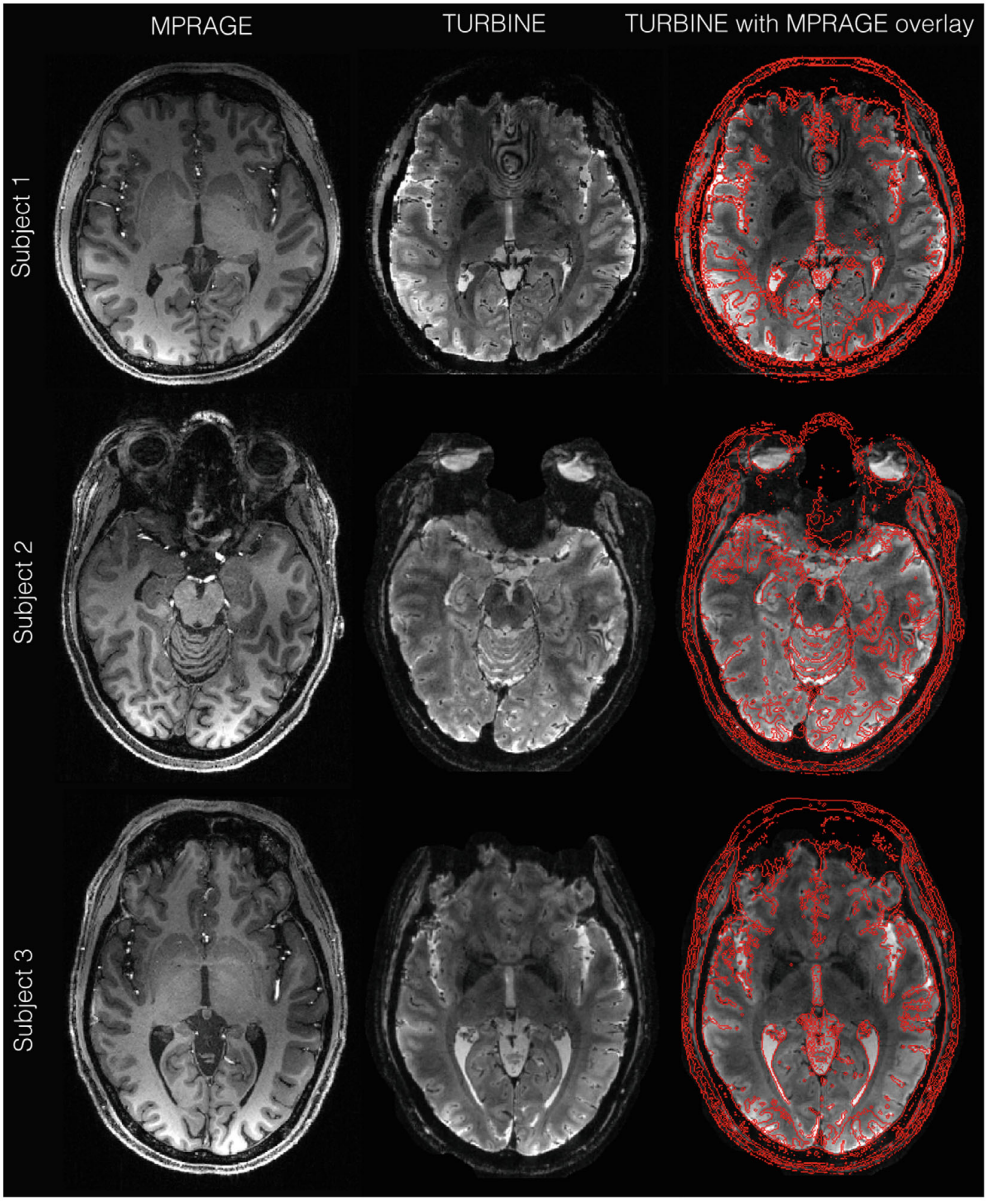
Structural MPRAGE image, TURBINE image (mean functional image), and MPRAGE contours overlaid on TURBINE image

Figure 3 shows the isotropic slab T_2_*‐weighted TURBINE data from 3 subjects over the visual cortex region, alongside the T_1_‐MPRAGE structural image highlighting the structural fidelity of the TURBINE data in the *x*‐*y* plane. The TURBINE image agrees very well with the red MPRAGE outline. There are only minor differences in the brain outline, likely due to dropout and/or small amounts of distortion in frontal areas, where the B_0_ field gradients are very high.

**FIGURE 4:**
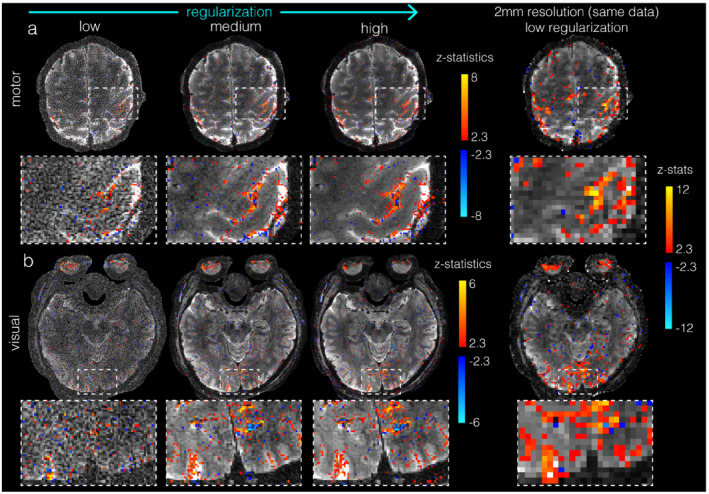
Impact of temporal regularization: 0.67 mm isotropic slab data set with motor (A) and visual (B) fMRI for 3 different regularization factors: very low, medium, and high. Right column shows same data reconstructed with 2 mm in‐plane resolution

Figure 4 shows the impact of the temporal regularization on the 0.67 mm isotropic TURBINE data. At $\lambda =10^{3}$, the low intrinsic SNR associated with the high‐resolution voxels is evident. At $\lambda =10^{4}$, the images are less noisy, but there is a small amount of residual streaking artifacts resulting from the relatively high radial acceleration factor. However, at $\lambda =10^{5}$, more temporal degrees of freedom are traded for improved spatial image fidelity, and the resulting images have no apparent radial aliasing artifacts. Therefore, all subsequent reconstructed data are shown at $\lambda =10^{5}$. As a comparison, the acquired data are also reconstructed at a lower resolution (2 mm) with low regularization, which shows consistent image contrast and BOLD activation with the high‐resolution datasets but with much poorer spatial specificity.

**FIGURE 5:**
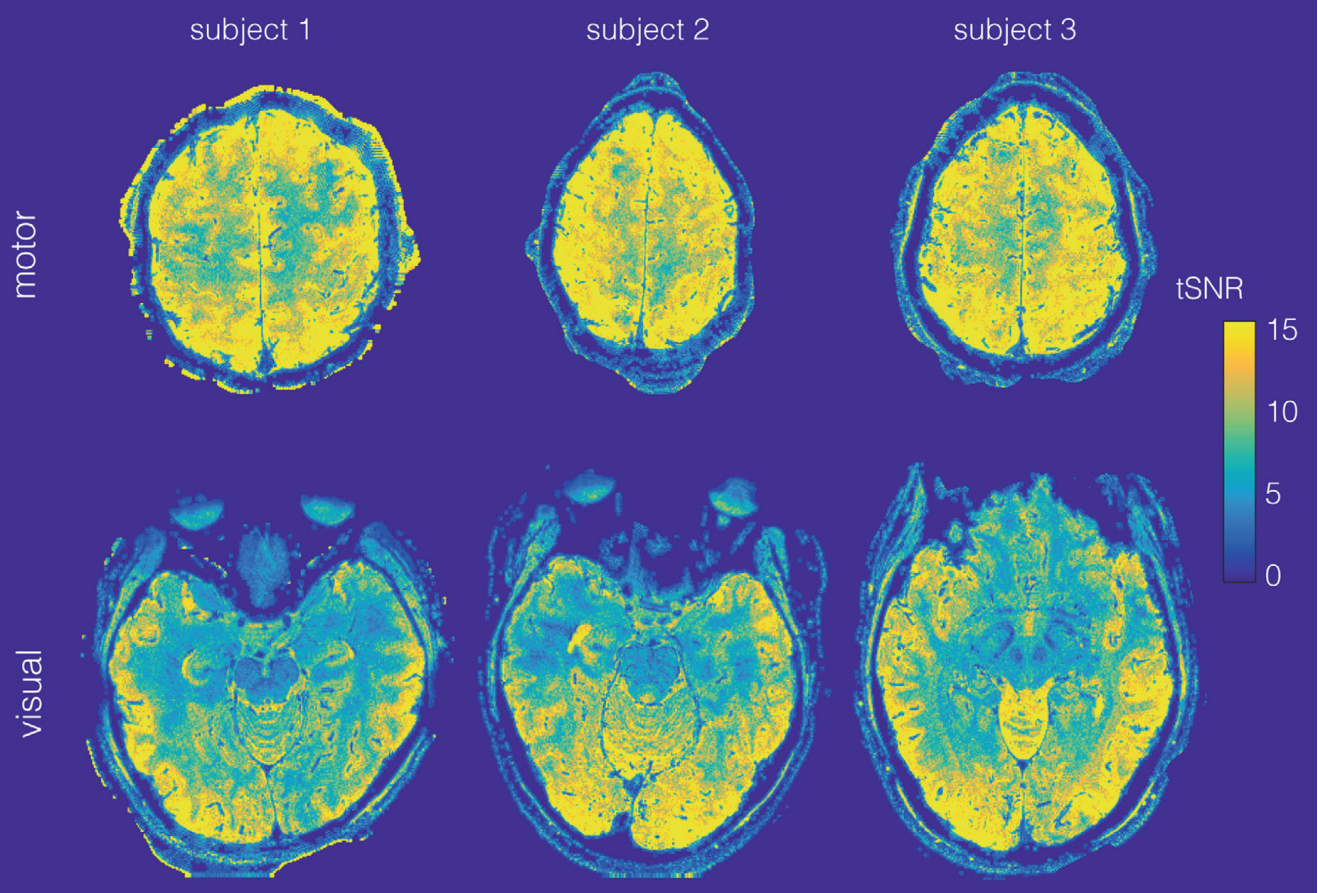
Maps of tSNR for all 6 slab fMRI data sets. Example slices (same slices as for fMRI results shown in Figure 7) for motor (top) and visual (bottom) slab data tSNR, temporal SNR.

To maximize BOLD sensitivity, all subsequent results were generated using the high regularization factor, including the DOF‐adjusted tSNR results shown in Figure 5. Average tSNR (using all brain voxels in the slab) was 11.6/12.9/13.0 for the motor slabs and 8.5/8.8/9.7 for the visual slabs for subjects 1/2/3, respectively.

**FIGURE 6:**
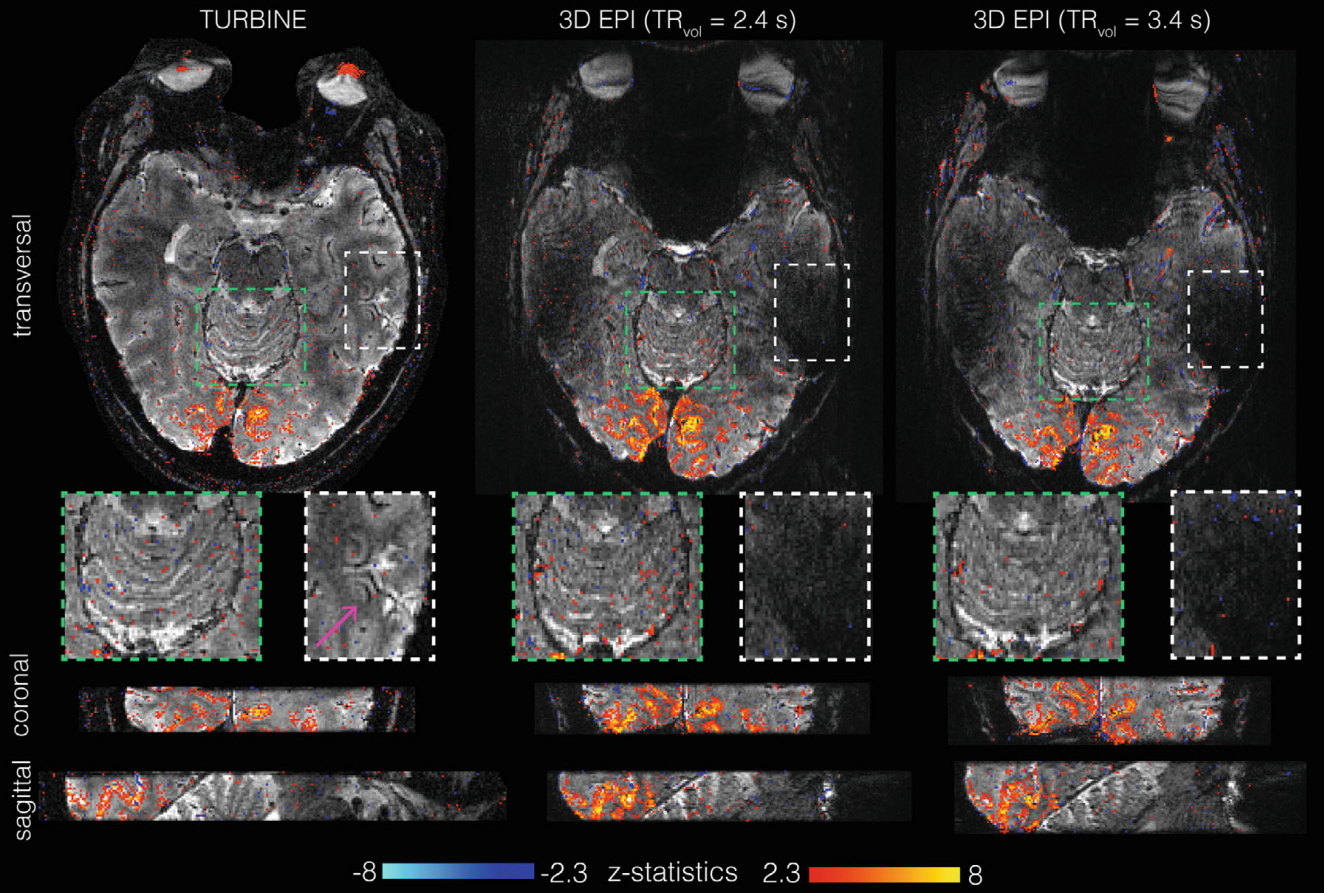
Qualitative comparison of TURBINE with 2 comparable 3D EPI protocols. Example functional image with *z*‐statistic maps overlaid in transversal, coronal, and sagittal view. Insets highlight resolution differences in the cerebellum (green box) and artifacts in the temporal lobes (white box)

A qualitative comparison between 3D EPI and TURBINE data acquired in a slab centered on the visual cortex is shown in Figure 6, with matched nominal spatial resolution. One EPI protocol had a matched nominal TR (2400 ms), and the second had a larger slab thickness for higher SNR, with a higher nominal TR (3400 ms). Both 3D EPI datasets show similar characteristics, expected of an EPI acquisition: signal dropout above the ear canals, spatial distortion, and blurring in the phase‐encode (anterior‐posterior) direction. In contrast, the TURBINE images do not exhibit any dropout, despite using the same B_0_‐shim values, and are blurring‐free and distortion‐free in the transversal plane. Distortion in the superior‐inferior direction is minimal due to the very short EPI train used in thin slab TURBINE acquisitions. In some of the high B_0_ inhomogeneity regions, a localized fringe‐like artifact can be seen (white box/pink arrow on Figure 6) on the TURBINE images. In the same area on the 3D EPI image, there is signal dropout of much larger spatial extent than the TURBINE artifact. Factors potentially explaining the reduced dropout in the TURBINE image are the shorter TE and the fact that the EPI direction is superior‐inferior. Matching these 2 would not be possible or practical for the 3D EPI (requiring the use of partial Fourier and many partitions to cover the FOV, i.e., long volume acquisition time).

In the image inset showing the cerebellum (green box), more anatomical details are visible in the TURBINE image, indicating that the true spatial resolution in the anterior‐posterior direction is higher than for the 3D EPI. The 3D EPI data sets showed strong BOLD activation with higher *z*‐statistic values; the TURBINE data generally showed slightly lower *z*‐stats. The TURBINE statistical maps exhibit less extensive activation than the 3D EPI ones, suggesting higher sensitivity of 3D EPI.

**FIGURE 7:**
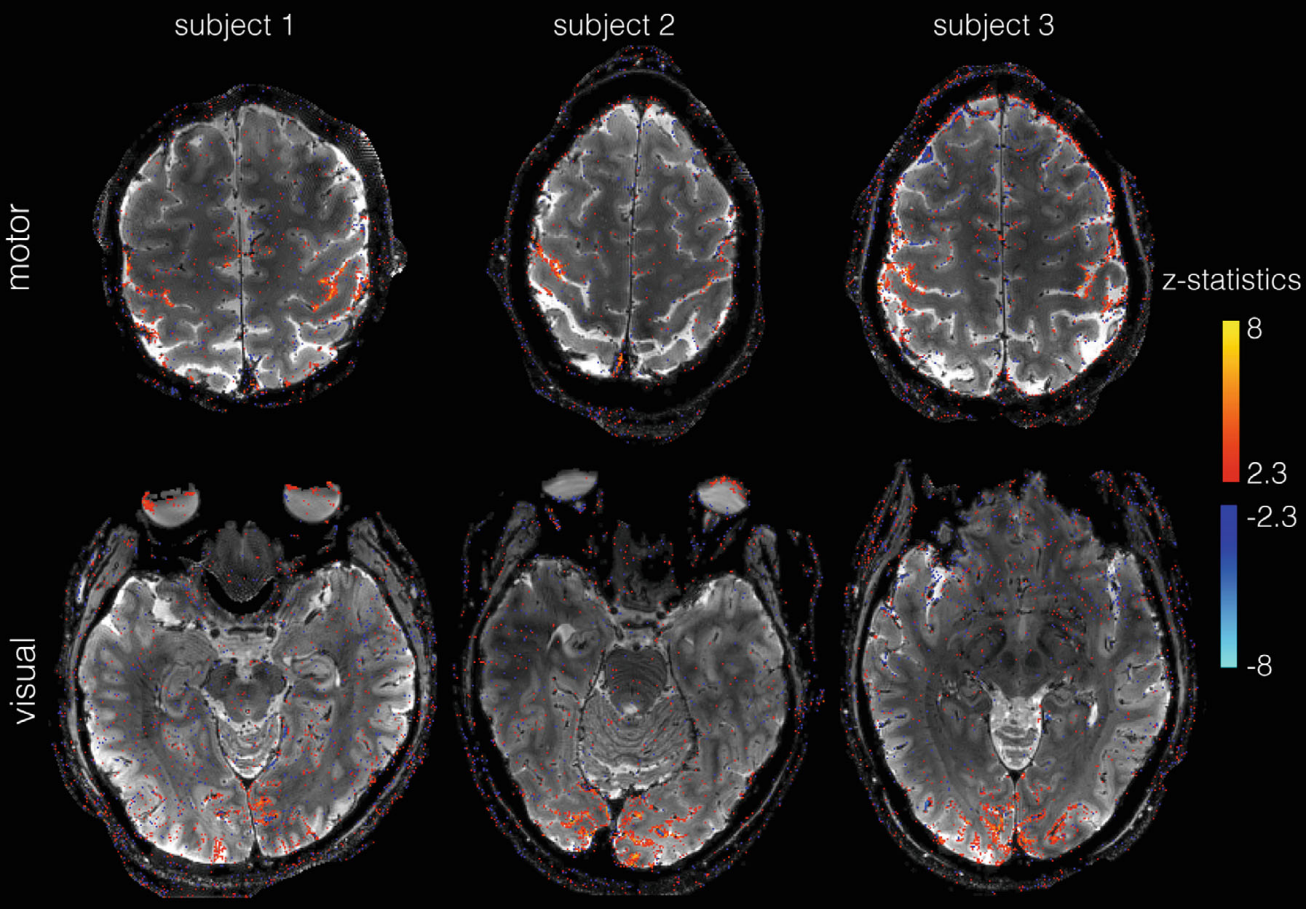
Activation maps for all high‐resolution (0.67 mm isotropic) slab data sets overlaid on mean functional TURBINE images. Top row shows motor (finger tapping) experiment for all 3 subjects; bottom shows visual (flashing checkerboard) experiment

Figure 7 shows activation maps for all 3 subjects, an example slice in the motor cortex in the top row, and an example visual cortex slice in the bottom row. For all data sets, there is activation in the expected areas of the motor and visual cortices. The voxels with significant z‐stats are localized to the gyri, indicating high spatial specificity.

**FIGURE 8:**
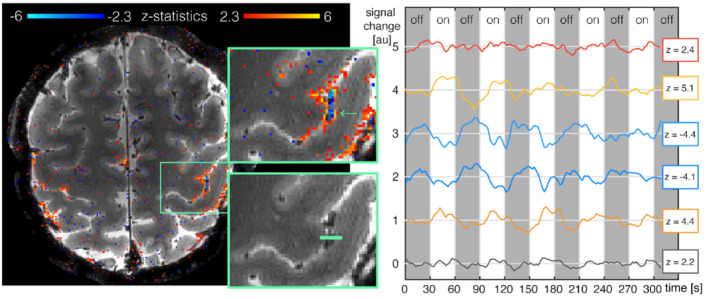
Motor activation map and time series of 0.67 mm isotropic fMRI. Insets with zoom on the primary motor cortex/central sulcus; bottom inset without activation maps shows row of 6 voxels (green) for which time‐course are shown on the right‐hand side. Demeaned time courses for the different voxels (separated by 1 au on the *y*‐axis) show progression from positive to negative *z*‐statistics and back to positive as we move across the central sulcus

To further illustrate the spatial fidelity of the acquired data, Figure 8 show a row of 6 voxels from the motor slab dataset, selected to lie across the central sulcus. From the voxel time‐courses, it can be seen that the outermost voxels on either side show little activation, with low *z*‐statistic values and no apparent task‐related modulation. Then, from left‐to‐right on the image (or from precentral to postcentral gyri), we have 1 voxel on the precentral gyrus showing positive BOLD modulation (*z* = 4.4); followed by 2 voxels that lie in the central sulcus showing negative BOLD fluctuation (z = −4.1 and −4.4, respectively); and finally, 1 voxel on the postcentral gyrus showing again strong positive BOLD signal (*z* = 5.1). This highlights the ability for the TURBINE data to capture submillimeter spatial variations in BOLD response, with distinct pre‐ and postcentral positive BOLD voxels and an interestingly strong negative BOLD response in the sulcus. The time‐courses in Figure 8 also indicate that the temporal regularization does not produce overly smooth time‐courses.

**FIGURE 9:**
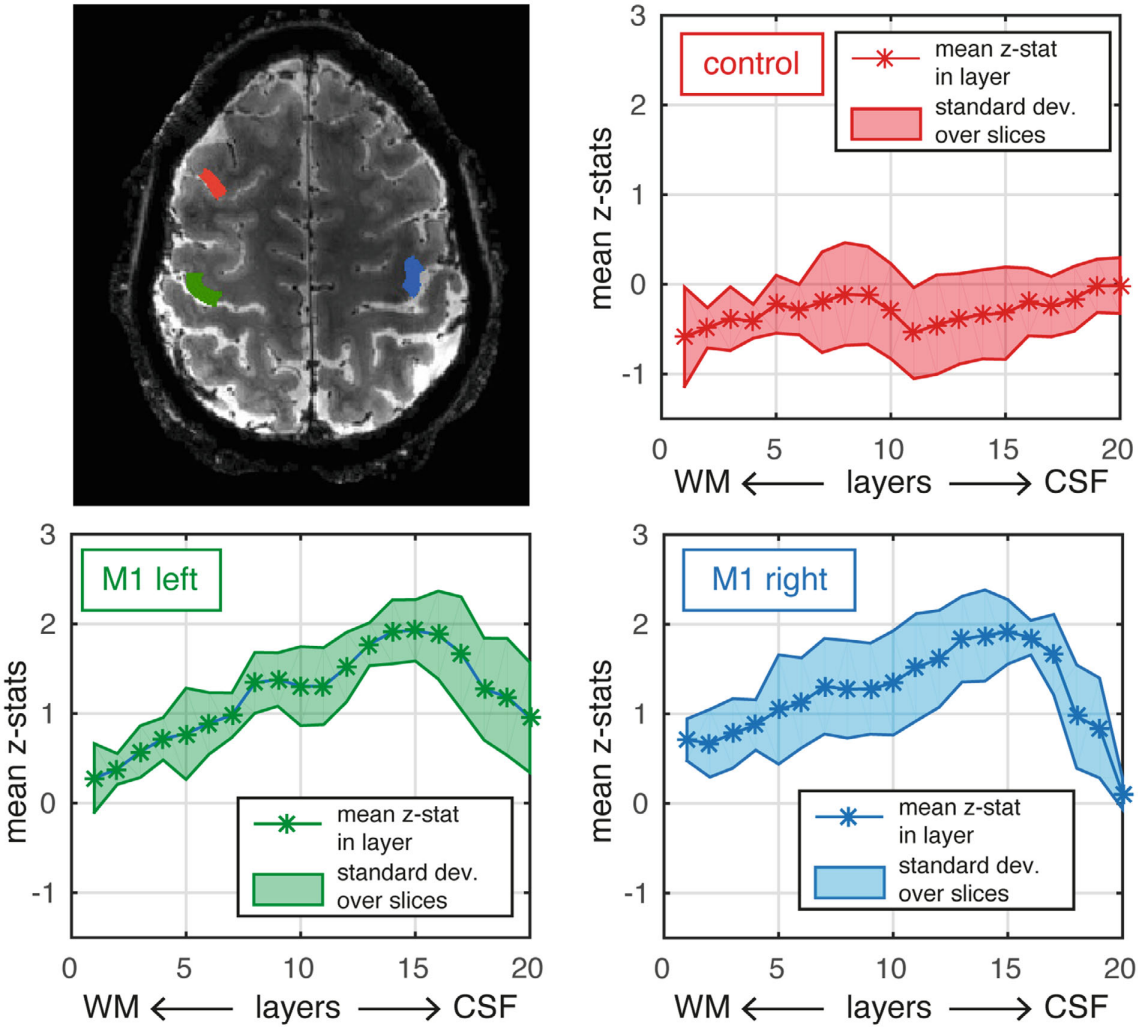
Layer‐specific analysis of 0.67 mm isotropic motor slab data. Top left shows mean functional TURBINE image, with 3 ROIs used for layer‐specific analysis. Hand area in left/right motor cortices (green/blue) and a control ROI (red) in gray matter area not expected to be activated by the task. The mean *z*‐stat values for all voxels in a layer are plotted. The shaded area corresponds to +/− the SD over the 4 slices used in the layer‐specific analysis.

To further investigate the usefulness of TURBINE fMRI for high‐resolution fMRI applications, we performed a layer‐specific analysis on the 0.67‐mm isotropic motor data for 1 subject. In Figure 9, line plots show the signal as a function of cortical depth for 20 layers between white matter and CSF. The data from the hand areas in M1 shows the expected bias to the pial surface, as expected in BOLD fMRI. The control ROI does not share this trend.

**FIGURE 10:**
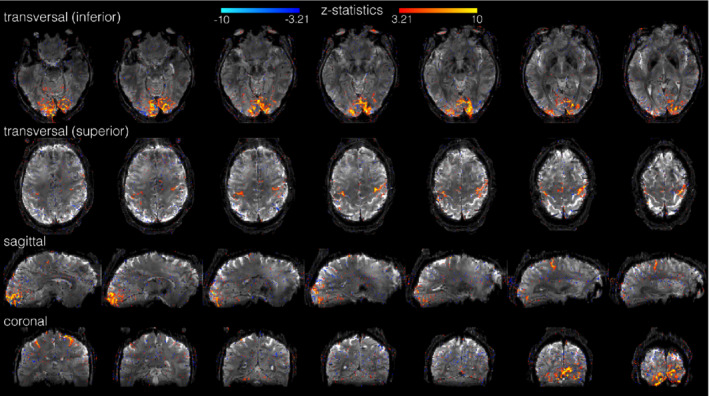
Whole‐brain task data (0.8 × 0.8 × 2 mm resolution) on 1 subject performing a simultaneous motor/visual task. Z‐statistic maps are overlaid on the mean functional TURBINE image. Top row shows inferior slices with visual activation, second row superior slices with motor activation. The bottom two rows display sagittal and coronal views respectively

Figure 10 shows images corresponding to the whole‐brain anisotropic TURBINE data from a single subject. These demonstrate that the TURBINE acquisition can be used to acquire data that is highly resolved in the radial plane (transverse here) but trading off reduced resolution in the phase‐encoding direction for whole brain coverage. However, the benefits of the TURBINE scheme are somewhat reduced because of the more significant impact of EPI‐like artifacts (dropout, distortion, and blurring) due to the longer echo trains required for whole‐brain coverage. One particularly notable artifact is most prominently visible in the transverse view, which appears like a localized shadow or reduction in signal intensity. Visualizations of the whole‐brain data of the second subject can be found in the Supporting Information, Figure S1.

## 4 DISCUSSION

In this work, we demonstrated the utility of hybrid radial‐Cartesian TURBINE sampling for minimally distorted, ultrahigh resolution fMRI at 7T. In conventional EPI, the in‐plane phase‐encoding direction is often oriented in the anterior‐posterior direction, that is, along the longest axis of the brain, typically to avoid asymmetric distortions between hemispheres. However, this results in long echo trains and consequently high distortion and T_2_* blurring in this direction. Using TURBINE, the EPI phase‐encoding direction is along the slab direction (here, superior‐inferior); consequently, the echo train is substantially shorter, even for whole‐brain coverage, and can be kept very short for thin slab acquisitions. The shorter TEs achievable with the shorter echo‐train also facilitates optimal BOLD contrast at 7T without requiring partial Fourier sampling. The TURBINE data acquired in this study was virtually distortion‐free in the transverse (radial) plane and showed excellent spatial correspondence to the T_1_ MPRAGE structural scan. Furthermore, it exhibited far fewer evident artifacts than the conventional multi‐shot 3D EPI data, which included visible distortion, dropout, and T_2_* blurring.

Although SNR‐efficient, a drawback of 3D multi‐shot imaging is the increased sensitivity to motion, physiological noise, and other fluctuations,^39^ which affects both TURBINE and Cartesian 3D EPI. However, due to the golden angle radial sampling pattern, TURBINE has integrated capabilities for artifact mitigation. Because a central k‐space column is repeatedly sampled with each readout, shot‐to‐shot global phase fluctuations (e.g., induced by respiration) can be corrected prior to image reconstruction by phase alignment of the k‐space origin for each shot. Integrated rigid body motion correction has also been demonstrated using the TURBINE acquisition scheme at 3T,^19^ but it was not used in this study. Initial work (not shown) has found motion correction to be less robust at 7T, likely due to more significant motion interactions with the B_0_ field, distortion effects, and image phase. Furthermore, to preserve the intrinsic resolution of the images, no post hoc motion correction was applied to the image time‐series, which is often a necessary preprocessing step in fMRI. We found that in compliant participants any benefits of motion correction were outweighed by image blurring resulting from the interpolation and resampling of standard motion correction schemes. However, motion‐correction procedures could be optimized to minimize blurring for future integration with TURBINE acquisition schemes.

A specific drawback of radial sampling schemes is that they are less efficient than Cartesian sampling,^40^ but the self‐navigating property and incoherent undersampling enabled by TURBINE at least in part compensates for the drop in raw SNR efficiency. The former property, alongside the phase corrections discussed above, also allows the readout to be self‐calibrating for the estimation of sensitivity maps or GRAPPA kernels. In this work, sensitivity maps were estimated from fully sampled images generated by binning all shots into a single temporal mean image. A useful feature of self‐calibration is the ability to generate dynamic calibration data where sudden movement might invalidate calibration data acquired before an experiment. The incoherence property of TURBINE trajectories makes it useful for sparse and/or low‐rank reconstructions.^21^, ^41^, ^42^ However, in this work, to prioritize spatial resolution and fidelity, no spatial regularization was applied in image reconstruction. Instead, only a temporal finite difference constraint was used to retain SNR, given the low intrinsic SNR provided by the 0.67 mm isotropic voxel size. The temporally regularized reconstruction does trade off effective temporal resolution, with approximately 0.40 effective temporal degrees of freedom retained from each nominal time‐point. However, despite the drop in effective time‐points, the smoothing regularized reconstruction results in a net benefit to statistical efficiency compared to an unregularized reconstruction.^31^ Because retention of SNR and tSNR is a key factor in imaging in ultrahigh resolution regimes with small voxels, exploring more sophisticated TURBINE‐like trajectories,^22^ regularization schemes,^43^ or denoising methods^44^ will be an interesting avenue of further study.

In a direct comparison, the TURBINE images did appear to have lower BOLD sensitivity than the matched 3D EPI data. Several factors could be contributing to this result. This includes T_2_* blurring in the 3D EPI data, in which sensitivity is increased (due to larger effective voxel size) at the cost of spatial specificity. Furthermore, the reduction of statistical degrees of freedom in the TURBINE data due to the regularized reconstruction could also be a contributing factor. However, because the proposed high‐resolution TURBINE sampling scheme was designed primarily to preserve spatial fidelity, the benefits of reduced distortion, blurring, and dropout should be balanced against the reduced sensitivity. The TURBINE trajectory is a viable alternative to 3D EPI in certain contexts, particularly when brain regions of interest such as frontal or temporal areas exhibit dropout or significant distortion, and 3D EPI is unable to provide robust signal or BOLD contrast. TURBINE should also provide higher effective spatial resolution compared to nominally matched 3D EPI readouts, which is consistent with qualitative inspection of the images, although a more comprehensive study of spatial point‐spread functions would be necessary to validate this observation. Furthermore, fidelity of brain structure means that conventional T_1_‐weighted structural images can be used in applications such as layer‐specific fMRI, rather than requiring a customized distortion‐matched T_1_‐weighted acquisition.

In the TURBINE images, phase artifacts in areas of rapidly changing B_0_ (e.g., regions where dropout was apparent in the 3D EPI data) were apparent as a visible fringe artifact in the isotropic thin slab data resulting from the interaction of the TURBINE readout with the local B_0_ field inhomogeneity. In the whole‐brain data shading, artifacts are visible, also likely due to an interaction of the trajectory with local off‐resonance effects or phase cancellation due to physiological fluctuations. However, the TURBINE artifacts are considerably less destructive than the characteristic 3D EPI dropout in that the spatial extent of the artifact is reduced and the signal magnitude is non‐zero within the artifact region. Off‐resonance interactions with non‐Cartesian reconstructions can be mitigated by including B_0_ information into the forward model during iterative image reconstruction. However, this increases computational complexity and relies on high‐fidelity field map information, and it was not utilized in the current work. The whole‐brain TURBINE data also showed localized reductions in signal intensity, likely due to a combination of factors including insufficient shot‐to‐shot phase correction and the larger voxel thickness (2.0 mm compared to 0.67 mm) increasing through‐plane dephasing in the anisotropic whole‐brain acquisition. Additional simulations (Figure S4) demonstrate similar artifacts can be reproduced as a consequence of un‐corrected residual dynamic phase fluctuations, supporting this hypothesis. Currently, the reconstructed data uses a zeroth order correction for shot‐to‐shot phase fluctuations based on the repeated sampling of the central column of k‐space. Future work may explore spatially resolved corrections based on this 1D navigator information for improved robustness to phase variations.

An interesting feature observed in the isotropic 0.67 mm TURBINE data was the apparent negative BOLD signal within the central sulcus, in between clearly separated positive BOLD responses on the gyri of the precentral M1 and postcentral S1. Figure S2 shows a similar pattern of positive BOLD response in visual cortex gyri, with correspondingly strong negative BOLD responses in the adjacent sulcus, ostensibly reproducing this phenomenon in a separate acquisition and brain region. One possible explanation for the negative sulcal signal could be the existence of a cerebral vein, for which evidence of negative BOLD signals has previously been observed.^45^ Very few studies in humans have been performed at this ultrahigh isotropic spatial resolution, with no in‐plane distortion or T_2_* blurring, which could explain why this particular spatial pattern of BOLD response has not been specifically reported in the literature. These preliminary observations require further investigation to determine the precise origin of the measured negative signals. For example, models of local tissue displacement have been shown to cause apparent negative signals in an fMRI acquisition.^46^ However, with increasing fidelity^37^ and resolution of fMRI in humans, we expect that more subtle spatiotemporal features, whether BOLD‐ or non‐BOLD–related, that are not detectable at conventional spatial resolutions will be revealed.

For finger‐tapping tasks that involved touching, as was performed in this work, a characteristic double peak pattern is expected in the M1‐4a subregion of the “hand‐knob” in M1.^47^ The superficial peak represents inputs from cortical‐to‐cortical connections (coming from the sensory areas due to the touch component of the task), whereas the secondary peak represents output activity via cortico‐spinal connections.^37^ In the laminar fMRI analysis of our data, we observed a strong bias toward the superficial layers. This is expected because the TURBINE method uses a T_2_*‐weighted gradient‐echo acquisition, draining veins at the cortical surface makes the laminar origin of gradient‐echoBOLD signals more ambiguous. Furthermore, partial‐volume effects, including voxels at the pial surface that have significant CSF contributions, will also bias the fMRI signals.^18^ However, because the TURBINE images display significantly reduced distortion and blurring compared to EPI, we expect partial volume effects to be reduced in the TURBINE data, particularly in regions where the pial boundary is perpendicular to the EPI phase‐encoding direction. Despite the bias, the suggestion of a double peak pattern was observed, and the overall findings were consistent with previously reported gradient‐echoBOLD laminar motor activity (e.g., Figure 3 in Ref. 37), indicating the utility of TURBINE acquisitions for laminar fMRI. In this work, TURBINE was employed for gradient‐echo BOLD, but the sampling scheme could also be used for other fMRI contrast mechanisms such as spin‐echo BOLD orVascular Space Occupancy.

## 5 CONCLUSION

TURBINE is a promising alternative to standard 3D EPI for ultrahigh‐resolution fMRI. It comes with the challenge of lower intrinsic sampling efficiency and a more time‐consuming image reconstruction but has the advantage of high spatial fidelity, low levels of artifact, and low T_2_* blurring. TURBINE could be beneficial for studies in areas that are particularly affected by B_0_ field inhomogeneity and when anatomical fidelity is crucial. The self‐navigated and incoherent sampling pattern make TURBINE a natural match with advanced artifact‐correction and image‐reconstruction schemes.

## FUNDING INFORMATION

The Wellcome Centre for Integrative Neuroimaging is supported by core funding from the Wellcome Trust (203139/Z/16/Z); and the Wellcome Centre for Human Neuroimaging is supported by core funding from the Wellcome Trust (203147/Z/16/Z).

K.L.M. is funded by a Wellcome Trust Senior Research Fellowship (202788/Z/16/Z). M.C. is funded by the Royal Academy of Engineering (RF201617∖16∖23) and the EPSRC (EP/T013133/1).

## Supporting information


**Figure S1.** Whole‐brain task data (0.8 × 0.8 × 2 mm resolution) of a second subject (see Figure 10 for the other subject) performing a simultaneous motor/visual task. Z‐statistic maps are overlaid on the mean functional TURBINE image. Top row shows inferior slices with visual activation, second row superior slices with motor activation. The bottom two rows display sagittal and coronal views respectively
**Figure S2.** Examples of negative BOLD signal in visual cortex sulci (as shown for motor cortex in Figure 8)
**Figure S3.** Radial sampling PSF (top), as well as “effective” PSF (bottom) demonstrating the impact and benefit of the temporal regularization on image quality. These PSFs do not include the encoding power of the coil sensitivities, and do not directly relate to the output image quality
**Figure S4.** Simulated dynamic field fluctuations, and using only a zeroth order phase correction prior to image reconstruction. In a numerical phantom, field fluctuations of ±5.0 and ± 10.0 Hz were compared to the ground truth. At ±10.0 Hz, we see the appearance of phase‐related signal cancellation artifacts (see for areas pointed out by red arrows), consistent with those appearing in the whole‐brain data
**Figure S5.** Highlighting retrospective motion correction challenges on thin slab data when using standard pipelines. Temporal standard deviation images, temporal mean images, tSNR and z‐statistic maps using three different pipelines: (1) default settings retrospective motion correction (MCFLIRT, FSL), (2) default settings after cropping volume to remove slices with signal drop‐off (slab profile effect) at the edges of the slab and (3) no motion correction. Temporal standard deviation images show high values around edges indicating there is motion in the time‐series whereas the image with no motion correction shows very little intensity increase around edges. However, the temporal standard deviation is lower in the motion corrected images in areas of homogenous signal. Visual inspection of the time‐series data suggests that the motion correction introduces small amounts of erroneous motion into this very low motion time series. The interpolation used additionally introduces smoothing which we believe leads to loss in resolution and subsequently higher z‐stats. All results shown in the paper use the no motion pipeline as there was very little motion in the data (no motion visible by eye). The choice to omit motion correction as a post‐processing step for our data is not an assertion that motion correction is not possible in this data, but simply a reflection of the fact that we observed degraded apparent spatial resolution in the data following conventional motion correction with default parameters, which may not be well tuned for this ultra‐high isotropic resolution data

## Data Availability

The raw measurement data for the isotropic slab data sets (visual and motor) and example image reconstruction code are available on https://github.com/mchiew/highres_turbine_7T.

## References

[1] Poser BA , Koopmans PJ , Witzel T , Wald LL , Barth M . Three dimensional echo‐planar imaging at 7 Tesla. Neuroimage. 2010;51:261‐266. 20139009 10.1016/j.neuroimage.2010.01.108 PMC2853246

[2] Posse S , Ackley E , Mutihac R , et al. Enhancement of temporal resolution and BOLD sensitivity in real‐time fMRI using multi‐slab echo‐volumar imaging. Neuroimage. 2012;61:115‐130. 22398395 10.1016/j.neuroimage.2012.02.059 PMC3342442

[3] Moeller S , Yacoub E , Olman CA , et al. Multiband multislice GE‐EPI at 7 Tesla, with 16‐fold acceleration using partial parallel imaging with application to high spatial and temporal whole‐brain fMRI. Magn Reson Med. 2010;63:1144‐1153. 20432285 10.1002/mrm.22361 PMC2906244

[4] Setsompop K , Gagoski BA , Polimeni JR , Witzel T , Wedeen VJ , Wald LL . Blipped‐controlled aliasing in parallel imaging for simultaneous multislice echo planar imaging with reduced g‐factor penalty. Magn Reson Med. 2012;67:1210‐1224. 21858868 10.1002/mrm.23097 PMC3323676

[5] Kok P , Bains LJ , Van Mourik T , Norris DG , De Lange FP . Selective activation of the deep layers of the human primary visual cortex by top‐down feedback. Curr Biol. 2016;26:371‐376. 26832438 10.1016/j.cub.2015.12.038

[6] Huber L , Tse DHY , Wiggins CJ , et al. Ultra‐high resolution blood volume fMRI and BOLD fMRI in humans at 9.4 T ‐ capabilities and challenges. Neuroimage. 2018;178:769‐779. 29890330 10.1016/j.neuroimage.2018.06.025 PMC6100753

[7] Finn ES , Huber L , Jangraw DC , Molfese PJ , Bandettini PA . Layer‐dependent activity in human prefrontal cortex during working memory. Nat Neurosci. 2019;22:1687‐1695. 31551596 10.1038/s41593-019-0487-z PMC6764601

[8] Zaitsev M , Zilles K , Shah NJ . Shared κ‐space echo planar imaging with keyhole. Magn Reson Med. 2001;45:109‐117. 11146492 10.1002/1522-2594(200101)45:1<109::aid-mrm1015>3.0.co;2-x

[9] Glover GH , Lee AT . Motion artifacts in fMRI: comparison of 2DFT with PR and spiral scan methods. Magn Reson Med. 1995;33:624‐635. 7596266 10.1002/mrm.1910330507

[10] Hu Y , Glover GH . Three‐dimensional spiral technique for high‐resolution functional MRI. Magn Reson Med. 2007;58:947‐951. 17969117 10.1002/mrm.21328

[11] Assländer J , Zahneisen B , Hugger T , et al. Single shot whole brain imaging using spherical stack of spirals trajectories. Neuroimage. 2013;73:59‐70. 23384526 10.1016/j.neuroimage.2013.01.065

[12] Grotz T , Zahneisen B , Ella A , Zaitsev M , Hennig J . Fast functional brain imaging using constrained reconstruction based on regularization using arbitrary projections. Magn Reson Med. 2009;62:394‐405. 19526512 10.1002/mrm.22009

[13] Lee GR , Griswold MA , Tkach JA . Rapid 3D radial multi‐echo functional magnetic resonance imaging. Neuroimage. 2010;52:1428‐1443. 20452436 10.1016/j.neuroimage.2010.05.004

[14] Zahneisen B , Grotz T , Lee KJ , et al. Three‐dimensional MR‐encephalography: fast volumetric brain imaging using rosette trajectories. Magn Reson Med. 2011;65:1260‐1268. 21294154 10.1002/mrm.22711

[15] Zahneisen B , Hugger T , Lee KJ , et al. Single shot concentric shells trajectories for ultra fast fMRI. Magn Reson Med. 2012;68:484‐494. 22131236 10.1002/mrm.23256

[16] Kasper L , Engel M , Heinzle J , et al. Advances in spiral fMRI: a high‐resolution study with single‐shot acquisition. Neuroimage. 2022;246:118738. 34800666 10.1016/j.neuroimage.2021.118738

[17] Singh V , Pfeuffer J , Zhao T , Ress D . Evaluation of spiral acquisition variants for functional imaging of human superior colliculus at 3T field strength. Magn Reson Med. 2018;79:1931‐1940. 28736924 10.1002/mrm.26845 PMC5783788

[18] Kashyap S , Ivanov D , Havlicek M , Sengupta S , Poser BA , Uludağ K . Resolving laminar activation in human V1 using ultra‐high spatial resolution fMRI at 7T. Sci Rep. 2018;8:17063. 30459391 10.1038/s41598-018-35333-3 PMC6244001

[19] Graedel NN , McNab JA , Chiew M , Miller KL . Motion correction for functional MRI with three‐dimensional hybrid radial‐Cartesian EPI. Magn Reson Med. 2017;78:527‐540. 27604503 10.1002/mrm.26390 PMC5516130

[20] McNab JA , Gallichan D , Miller KL . 3D steady‐state diffusion‐weighted imaging with trajectory using radially batched internal navigator echoes (TURBINE). Magn Reson Med. 2010;63:235‐242. 19859953 10.1002/mrm.22183

[21] Chiew M , Graedel NN , McNab JA , Smith SM , Miller KL . Accelerating functional MRI using fixed‐rank approximations and radial‐cartesian sampling. Magn Reson Med. 2016;76:1825‐1836. 26777798 10.1002/mrm.26079 PMC4847647

[22] Rettenmeier CA , Maziero D , Stenger VA . Three dimensional radial echo planar imaging for functional MRI. Magn Reson Med. 2022;87:193‐206. 34411342 10.1002/mrm.28980 PMC8616809

[23] Stäb D , Bollmann S , Langkammer C , Bredies K , Barth M . Accelerated mapping of magnetic susceptibility using 3D planes‐on‐a‐paddlewheel (POP) EPI at ultra‐high field strength. NMR Biomed. 2017;30:e3620. 10.1002/nbm.3620 27763692

[24] Winkelmann S , Schaeffter T , Koehler T , Eggers H , Dössel O . An optimal radial profile order based on the golden ratio for time‐resolved MRI. IEEE Trans Med Imaging. 2007;26:68‐76. 17243585 10.1109/TMI.2006.885337

[25] Pruessmann KP , Weiger M , Scheidegger MB , Boesiger P . SENSE: sensitivity encoding for fast MRI. 1999;42:952‐962. 10542355

[26] Griswold MA , Jakob PM , Heidemann RM , et al. Generalized autocalibrating partially parallel acquisitions (GRAPPA). 2002;47:1202‐1210. 10.1002/mrm.10171 12111967

[27] Breuer FA , Blaimer M , Mueller MF , et al. Controlled aliasing in volumetric parallel imaging (2D CAIPIRINHA). Magn Reson Med. 2006;55:549‐556. 16408271 10.1002/mrm.20787

[28] Zur Y , Wood ML , Neuringer LJ . Spoiling of transverse magnetization in steady‐state sequences. Magn Reson Med. 1991;21:251‐263. 1745124 10.1002/mrm.1910210210

[29] Walsh DO , Gmitro AF , Marcellin MW . Adaptive reconstruction of phased array MR Imagery. Magn Reson Med. 2000;43:682‐690. 10800033 10.1002/(sici)1522-2594(200005)43:5<682::aid-mrm10>3.0.co;2-g

[30] Buehrer M , Pruessmann KP , Boesiger P , Kozerke S . Array compression for MRI with large coil arrays. Magn Reson Med. 2007;57:1131‐1139. 17534913 10.1002/mrm.21237

[31] Chiew M , Miller KL . Improved statistical efficiency of simultaneous multi‐slice fMRI by reconstruction with spatially adaptive temporal smoothing. Neuroimage. 2019;203:116165. 31494247 10.1016/j.neuroimage.2019.116165 PMC6854456

[32] Fessler JA , Sutton BP . Nonuniform fast Fourier transforms using min‐max interpolation. IEEE Trans Signal Process. 2003;51:560‐574.

[33] Friston KJ , Holmes AP , Poline JB , et al. Analysis of fMRI time‐series revisited. Neuroimage. 1995;2:45‐53. 9343589 10.1006/nimg.1995.1007

[34] Jenkinson M , Beckmann CF , Behrens TEJ , Woolrich MW , Smith SM . FSL. Neuroimage. 2012;62:782‐790. 21979382 10.1016/j.neuroimage.2011.09.015

[35] Beckmann CF , Smith SM . Probabilistic independent component analysis for functional magnetic resonance imaging. IEEE Trans Med Imaging. 2004;23:137‐152. 14964560 10.1109/TMI.2003.822821

[36] Feinberg DA , Moeller S , Smith SM , et al. Multiplexed echo planar imaging for sub‐second whole brain FMRI and fast diffusion imaging. PLoS One. 2010;5:e15710. 21187930 10.1371/journal.pone.0015710 PMC3004955

[37] Huber L , Handwerker DA , Jangraw DC , et al. High‐resolution CBV‐fMRI allows mapping of laminar activity and connectivity of cortical input and output in human M1. Neuron. 2017;96:1253‐1263.e7. 29224727 10.1016/j.neuron.2017.11.005 PMC5739950

[38] Huber L , Poser BA , Bandettini PA , et al. LayNii: a software suite for layer‐fMRI. Neuroimage. 2021;237:118091. 33991698 10.1016/j.neuroimage.2021.118091 PMC7615890

[39] van der Zwaag W , Marques JP , Kober T , Glover G , Gruetter R , Krueger G . Temporal SNR characteristics in segmented 3D‐EPI at 7T. Magn Reson Med. 2012;67:344‐352. 21656557 10.1002/mrm.23007 PMC3627735

[40] Tsai CM , Nishimura DG . Reduced aliasing artifacts using variable‐density k‐space sampling trajectories. Magn Reson Med. 2000;43:452‐458. 10725889 10.1002/(sici)1522-2594(200003)43:3<452::aid-mrm18>3.0.co;2-b

[41] Weizman L , Miller KL , Eldar YC , Maayan O , Chiew M . PEAR: Periodic and aperiodic signal separation for fast FMRI. Annu Int Conf IEEE Eng Med Biol Soc. 2017;2017:505‐508. 29059920 10.1109/EMBC.2017.8036872

[42] Chiew M , Graedel NN , Miller KL . Recovering task fMRI signals from highly under‐sampled data with low‐rank and temporal subspace constraints. Neuroimage. 2018;174:97‐110. 29501875 10.1016/j.neuroimage.2018.02.062 PMC5953310

[43] Mason HT , Graedel NN , Miller KL , Chiew M . Subspace‐constrained approaches to low‐rank fMRI acceleration. Neuroimage. 2021;238:118235. 34091032 10.1016/j.neuroimage.2021.118235 PMC7611820

[44] Moeller S , Pisharady PK , Ramanna S , et al. NOise Reduction with DIstribution Corrected (NORDIC) PCA in dMRI with complex‐valued parameter‐free locally low‐rank processing. Neuroimage. 2021;226:117539. 33186723 10.1016/j.neuroimage.2020.117539 PMC7881933

[45] Bianciardi M , Fukunaga M , Van Gelderen P , De Zwart JA , Duyn JH . Negative BOLD‐fMRI signals in large cerebral veins. J Cereb Blood Flow Metab. 2011;31:401‐412. 20859295 10.1038/jcbfm.2010.164 PMC3049531

[46] Zoraghi M , Scherf N , Jaeger C , et al. Simulating local deformations in the human cortex due to blood flow‐induced changes in mechanical tissue properties: impact on functional magnetic resonance imaging. Front Neurosci. 2021;15:722366. 34621151 10.3389/fnins.2021.722366 PMC8490675

[47] Terumitsu M , Ikeda K , Kwee IL , Nakada T . Participation of primary motor cortex area 4a in complex sensory processing: 3.0‐T fMRI study. Neuroreport. 2009;20:679‐683. 19339906 10.1097/WNR.0b013e32832a1820

