# Supplementary material for: Ultrahigh Resolution fMRI at 7T Using Radial‐Cartesian TURBINE Sampling
Source: Magn Reson Med. 2022 Jul 4;88(5):2058–73. doi: 10.1002/mrm.29359 (PMC9546489; doi:10.1002/mrm.29359)
Supplement: Supplementary file 1 — Figure S1. Whole‐brain task data (0.8 × 0.8 × 2 mm resolution) of a second subject (see Figure 10 for the other subject) performing a simultaneous motor/visual task. Z‐statistic maps are overlaid on the mean functional TURBINE image. Top row shows inferior slices with visual activation, second row superior slices with motor activation. The bottom two rows display sagittal and coronal views respectively Figure S2. Examples of negative BOLD signal in visual cortex sulci (as shown for motor cortex in Figure 8) Figure S3. Radial sampling PSF (top), as well as “effective” PSF (bottom) demonstrating the impact and benefit of the temporal regularization on image quality. These PSFs do not include the encoding power of the coil sensitivities, and do not directly relate to the output image quality Figure S4. Simulated dynamic field fluctuations, and using only a zeroth order phase correction prior to image reconstruction. In a numerical phantom, field fluctuations of ±5.0 and ± 10.0 Hz were compared to the ground truth. At ±10.0 Hz, we see the appearance of phase‐related signal cancellation artifacts (see for areas pointed out by red arrows), consistent with those appearing in the whole‐brain data Figure S5. Highlighting retrospective motion correction challenges on thin slab data when using standard pipelines. Temporal standard deviation images, temporal mean images, tSNR and z‐statistic maps using three different pipelines: (1) default settings retrospective motion correction (MCFLIRT, FSL), (2) default settings after cropping volume to remove slices with signal drop‐off (slab profile effect) at the edges of the slab and (3) no motion correction. Temporal standard deviation images show high values around edges indicating there is motion in the time‐series whereas the image with no motion correction shows very little intensity increase around edges. However, the temporal standard deviation is lower in the motion corrected images in areas of homogenous signal. Visual inspect [file MRM-88-2058-s001.docx]

# Supporting information


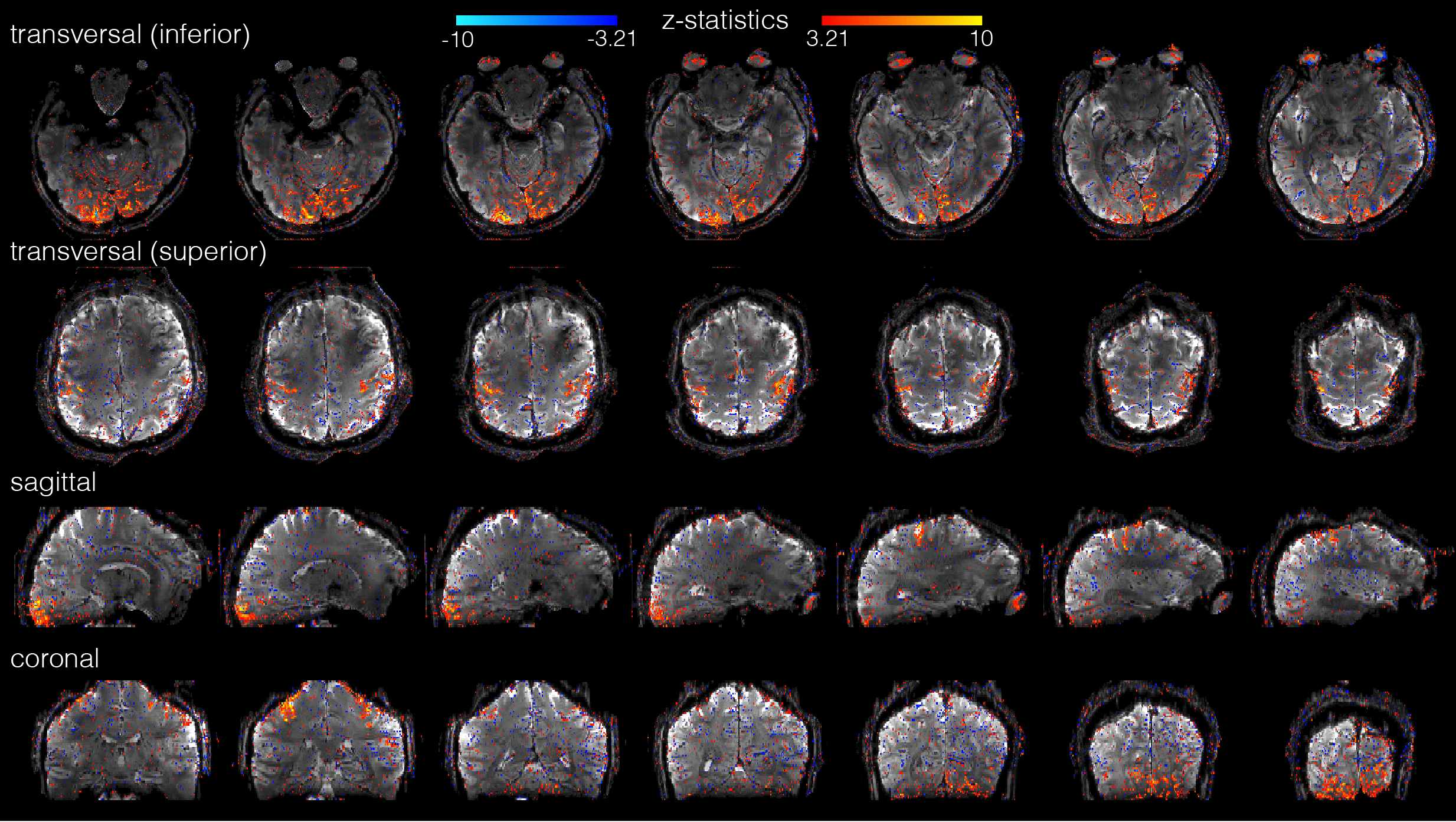


Supporting Figure S1 - Whole-brain task data (0.8x0.8x2 mm resolution) of a second subject (see Fig. 10 for the other subject) performing a simultaneous motor/visual task. Z-statistic maps are overlaid on the mean functional TURBINE image. Top row shows inferior slices with visual activation, second row superior slices with motor activation. The bottom two rows display sagittal and coronal views respectively.


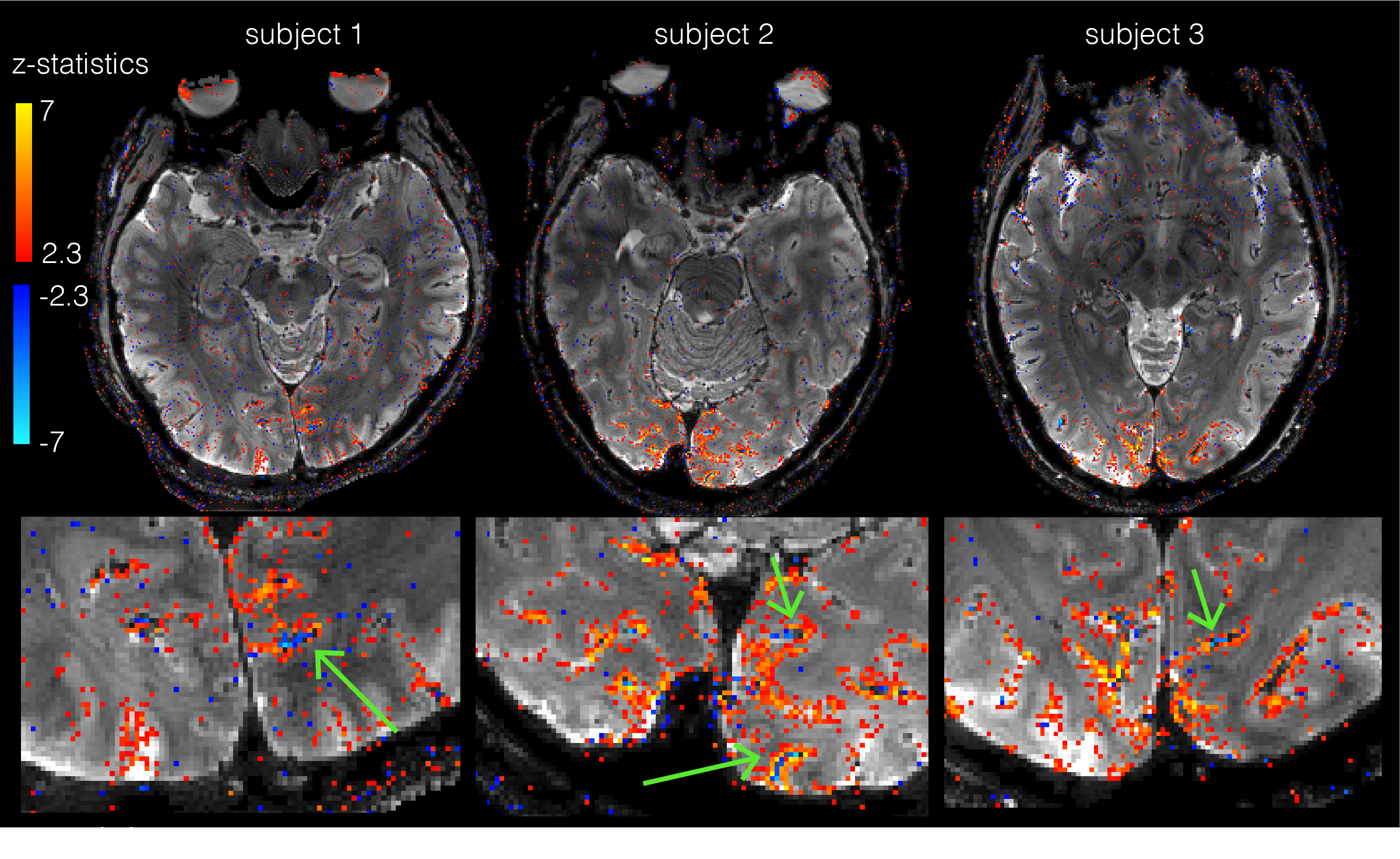


Supporting Figure S2 – Examples of negative BOLD signal in visual cortex sulci (as shown for motor cortex in Figure 8).


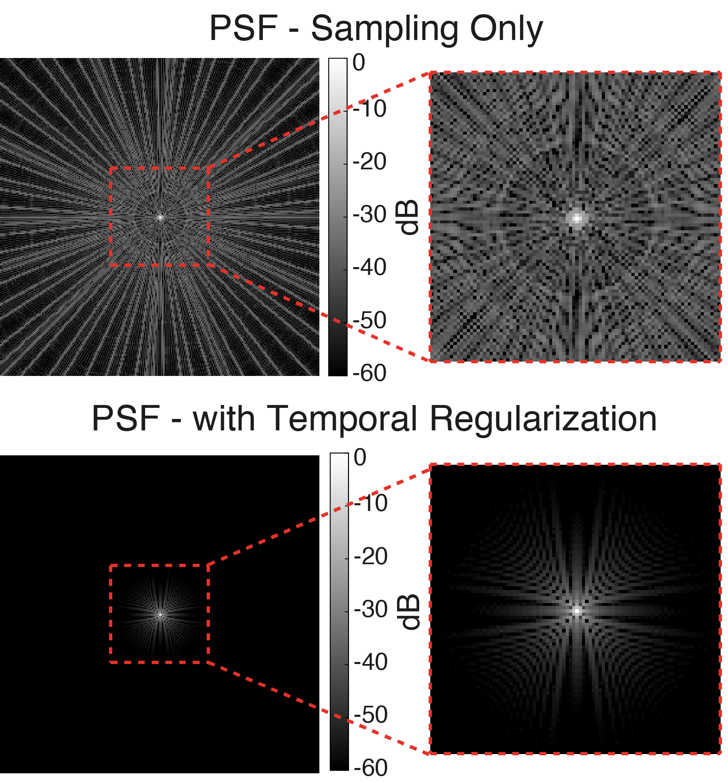


Supporting Figure S3 – radial sampling PSF (top), as well as "effective" PSF (bottom) demonstrating the impact and benefit of the temporal regularization on image quality. These PSFs do not include the encoding power of the coil sensitivities, and do not directly relate to the output image quality.


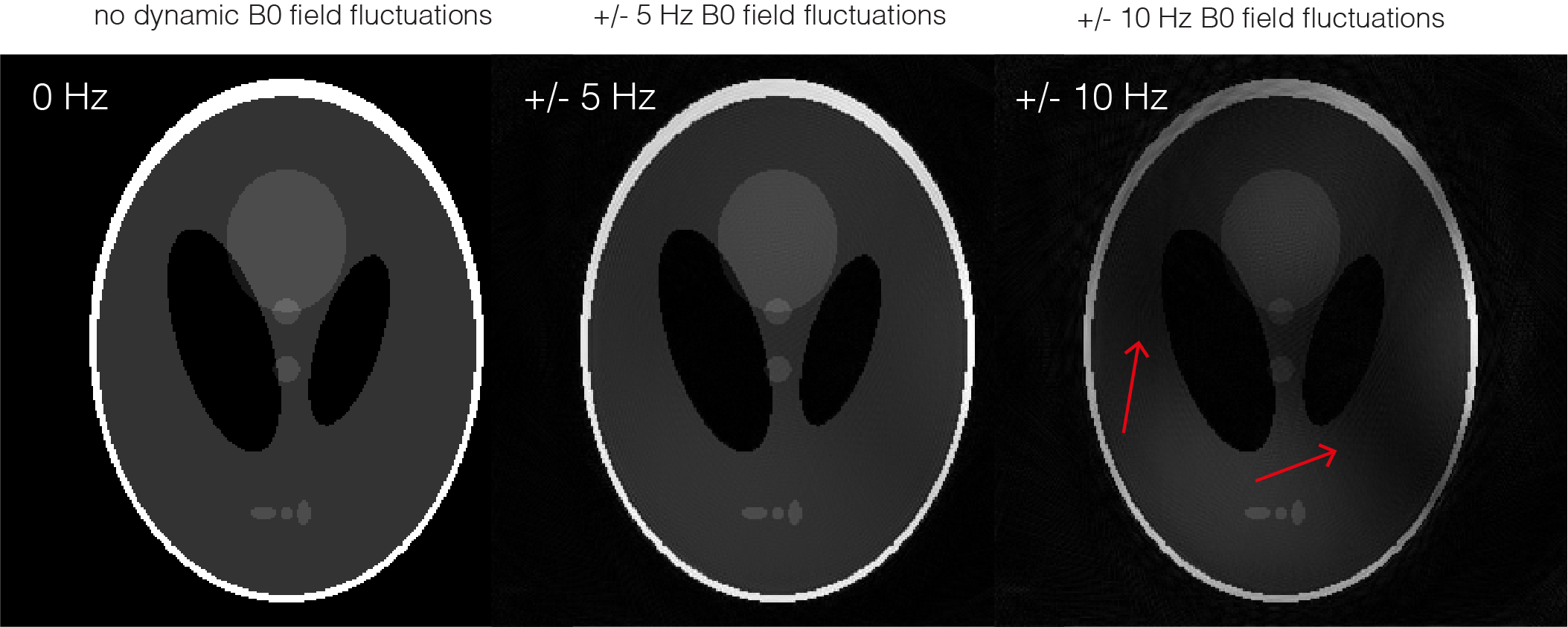


Supporting Figure S4 – Simulated dynamic field fluctuations, and using only a zeroth order phase correction prior to image reconstruction. In a numerical phantom, field fluctuations of ±5.0 and ±10.0 Hz were compared to the ground truth. At ±10.0 Hz, we see the appearance of phase-related signal cancellation artifacts (see for areas pointed out by red arrows), consistent with those appearing in the whole-brain data.


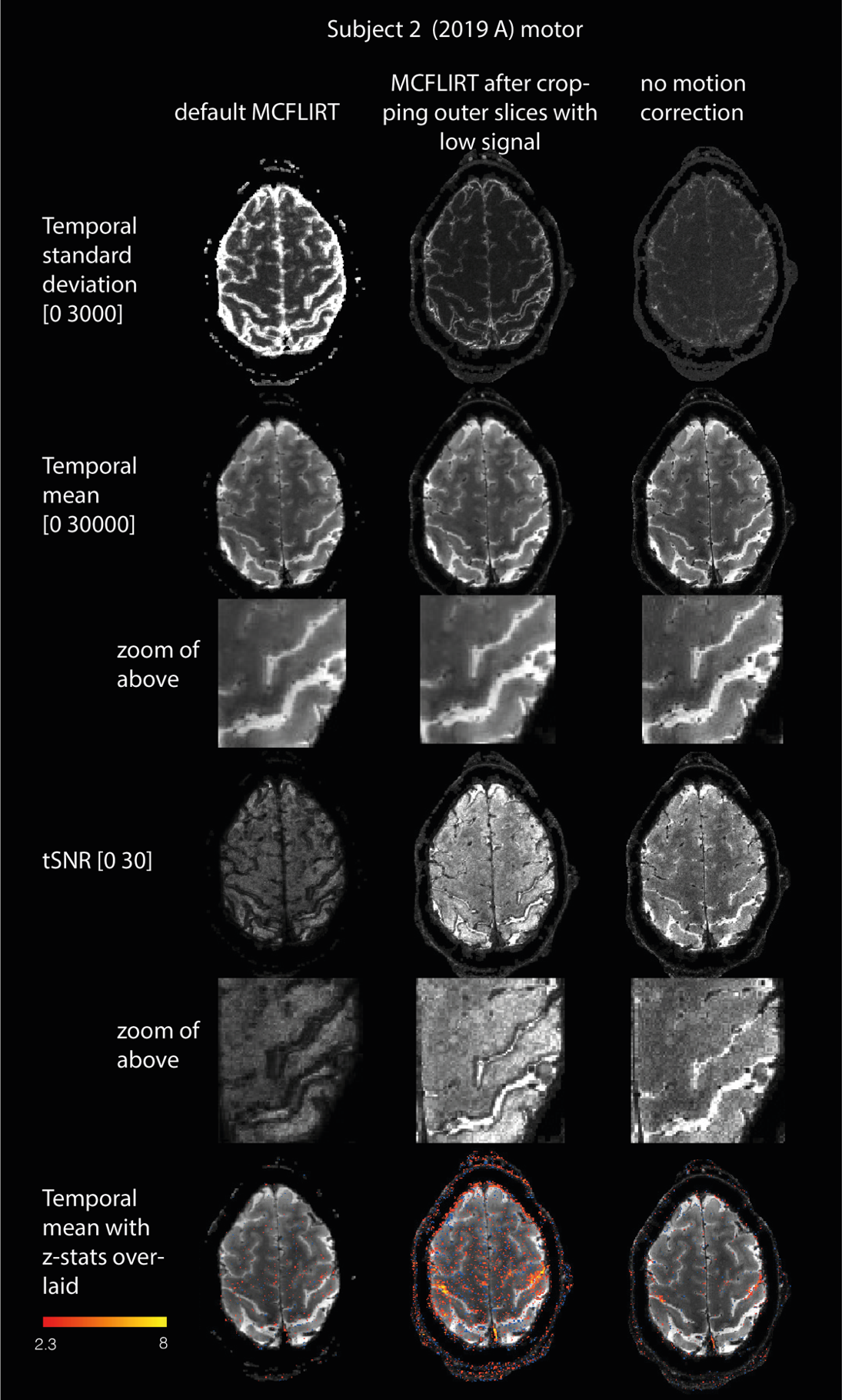


Supporting Figure S5 – Highlighting retrospective motion correction challenges on thin slab data when using standard pipelines. Temporal standard deviation images, temporal mean images, tSNR and z-statistic maps using three different pipelines: (1) default settings retrospective motion correction (MCFLIRT, FSL), (2) default settings after cropping volume to remove slices with signal drop-off (slab profile effect) at the edges of the slab and (3) no motion correction. Temporal standard deviation images show high values around edges indicating there is motion in the time-series whereas the image with no motion correction shows very little intensity increase around edges. However, the temporal standard deviation is lower in the motion corrected images in areas of homogenous signal. Visual inspection of the time-series data suggests that the motion correction introduces small amounts of erroneous motion into this very low motion time series. The interpolation used additionally introduces smoothing which we believe leads to loss in resolution and subsequently higher z-stats. All results shown in the paper use the no motion pipeline as there was very little motion in the data (no motion visible by eye). The choice to omit motion correction as a post-processing step for our data is not an assertion that motion correction is not possible in this data, but simply a reflection of the fact that we observed degraded apparent spatial resolution in the data following conventional motion correction with default parameters, which may not be well tuned for this ultra-high isotropic resolution data.
